# Arthroscopy-assisted treatment of giant cell tumor of the proximal femur: a case report

**DOI:** 10.3389/fsurg.2026.1774122

**Published:** 2026-03-31

**Authors:** Hao Chen, Ya Sun, Fei Gao, Bo Wang, Weiwei Hua

**Affiliations:** Department of Sports Medicine, The Chinese People’s Armed Police Forces Anhui Provincial Corps Hospital, Hefei, China

**Keywords:** case report, giant cell tumor, hip arthroscopy, mixed grafting, proximal femur

## Abstract

Giant cell tumor of bone (GCT) is a borderline primary bone tumor characterized by locally aggressive behavior, for which surgical management is the mainstay of treatment. Reports on GCT involving the proximal femur remain relatively limited. Achieving complete lesion removal while preserving hip joint function continues to represent a clinical challenge. A 25-year-old man presented with right hip pain. Imaging examinations revealed an occupying lesion in the proximal region of the right femur. The patient underwent lesion curettage and inactivation assisted by hip arthroscopy, followed by bone defect reconstruction using a combination of artificial bone and autologous bone grafting. This case suggests that arthroscopy-assisted lesion curettage combined with bone grafting may facilitate local tumor control while preserving hip joint function, and may serve as a reference for the management of similar cases.

## 1 Introduction

Giant cell tumor of bone (GCT) is a primary bone tumor characterized by locally aggressive behavior and most commonly arises in the epiphyseal region of long bones, with approximately 50%–65% of lesions occurring around the knee joint (1). Although the distal femur and proximal tibia are the most commonly involved sites, GCT occurring in the proximal femur is relatively uncommon (2). At present, for GCT without obvious soft tissue extension, intralesional curettage remains the primary treatment approach, aiming to achieve thorough tumor removal while preserving as much bone tissue as possible. When necessary, bone defects may be reconstructed using artificial bone or bone cement, which generally yields satisfactory clinical outcomes. In contrast, lesions with soft tissue involvement often require wide local resection (3). However, because the proximal femur is anatomically adjacent to the hip joint and plays a critical role in weight bearing, achieving complete lesion removal while minimizing local recurrence risk and preserving hip joint function remains particularly challenging, and optimal treatment strategies have yet to reach a clear consensus (4). Particularly in the field of minimally invasive surgery, reports on arthroscopy-assisted treatment for GCT of the proximal femur remain very limited, and further clinical experience is still needed.

## 2 Case presentation

**Figure 1:**
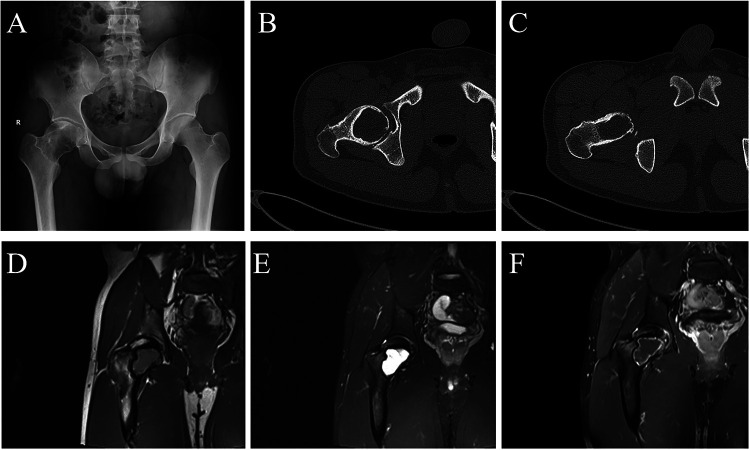
Preoperative imaging findings of the proximal femur. **(A)** DR showing a low-density lesion in the proximal femur. **(B,C)** CT demonstrating cystic low-density lesions involving the femoral head and neck, with marginal sclerosis. **(D–F)** MRI revealing an isointense signal on T1-weighted images and a hyperintense signal on T2-weighted images.

A 25-year-old Chinese male track athlete with a 5-year history of high-intensity hurdle training presented with right hip pain. The discomfort had begun 2 months earlier and did not improve with rest. One week before admission, the pain markedly worsened after training and progressively impaired ambulation, prompting him to seek medical attention. Physical examination revealed marked deep tenderness in the right inguinal region. Hip flexion was not significantly limited, whereas adduction and abduction were markedly restricted. The FABER test could not be completed because of pain provocation. Imaging examinations were subsequently performed. Digital radiography (DR) of the hip demonstrated decreased bone density of the right femoral head and neck, with thinning of the cortical bone at the lesion margin. Computed tomography (CT) revealed a cystic low-density lesion involving the proximal femur, including the femoral head and neck, with marginal sclerosis. Magnetic resonance imaging (MRI) showed an occupying lesion in the proximal femur with isointense signal on T1-weighted images and hyperintense signal on T2-weighted images, without obvious enhancement on contrast-enhanced sequences (Figure 1). Based on these findings, the patient underwent arthroscopy-assisted curettage of the proximal femoral lesion with mixed bone grafting.

**Figure 2:**
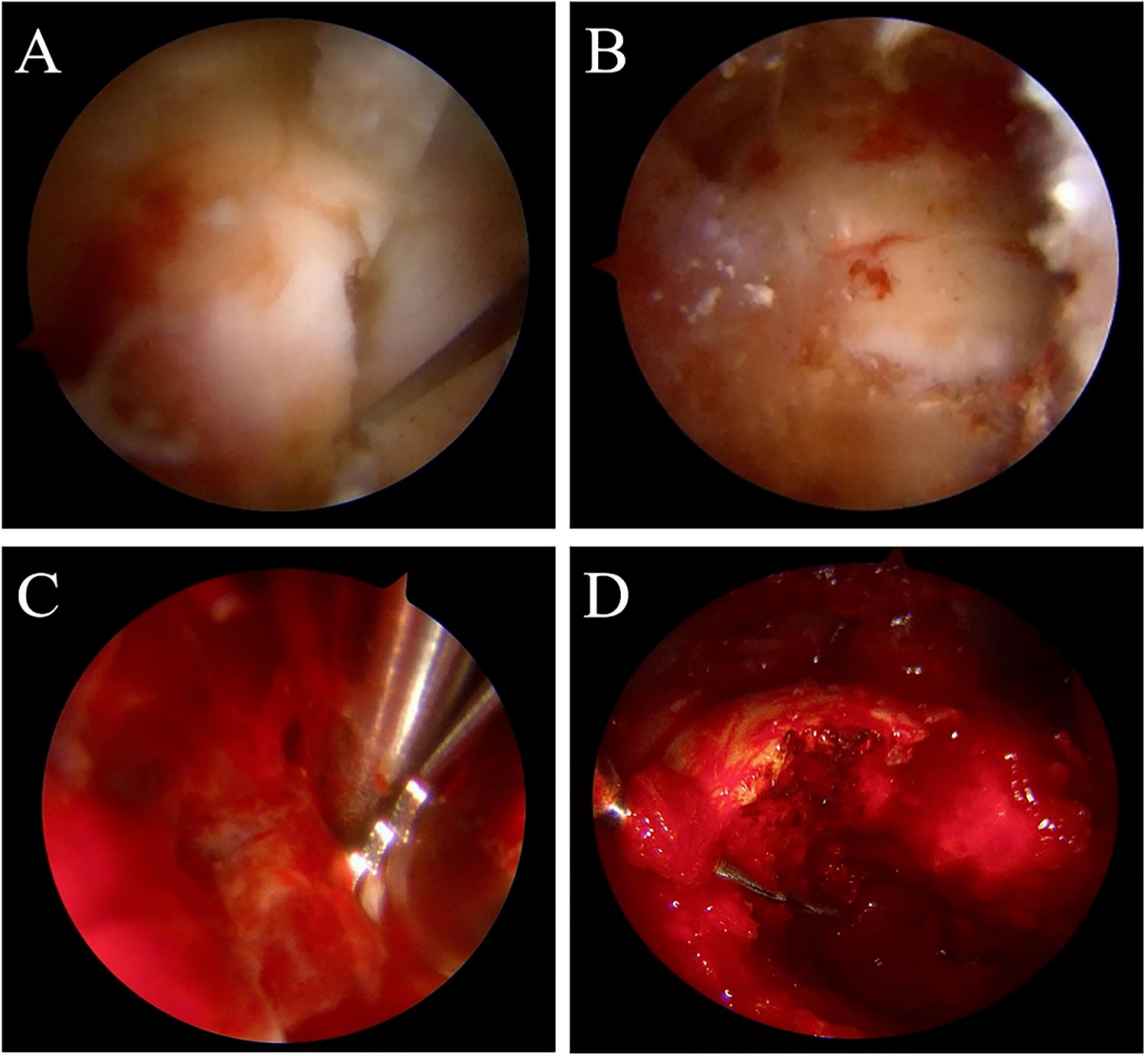
Intraoperative arthroscopic findings and bone grafting procedure. **(A)** Creation of a bone window at the lesion site under arthroscopic visualization. **(C)** The lesion cavity filled with yellow, mass-like tissue. **(D)** Irregular margins of the lesion cavity. **(B)** Repositioning of the bone window after completion of bone grafting.

**Figure 3:**
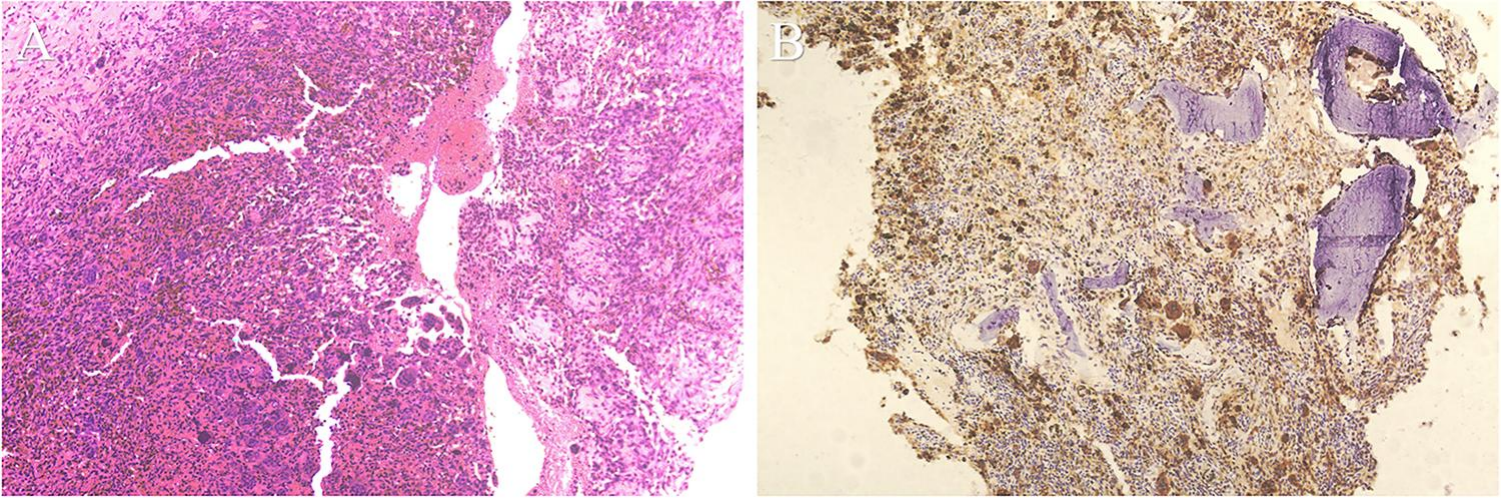
Histopathological findings of the lesion. The presence of multinucleated giant cells on histological examination **(A)**, together with CD68 positivity on immunohistochemistry **(B)**, supports the diagnosis of GCT.

Under hip arthroscopy assistance, the right hip joint capsule was incised, and a bone window was established at the anterolateral aspect of the right femoral neck under arthroscopic visualization. Intraoperative findings revealed a large cavitary lesion in the proximal femur, filled with pale yellow, mass-like solid tissue, with irregular lesion margins. After partial removal of the lesion for pathological examination, a shaver was introduced into the cavity for thorough curettage. Lesion inactivation was further performed using iodine injection and plasma radiofrequency ablation. Subsequently, autologous bone harvested from the ipsilateral iliac crest was mixed with artificial bone graft material and packed into the defect cavity. The bone window was firmly repositioned and stabilized. One all-suture anchor was placed adjacent to the bone window, and the joint capsule was repaired arthroscopically (Figure 2). Histopathological examination demonstrated diffuse proliferation of mononuclear tumor cells with scattered multinucleated giant cells, consistent with GCT. Immunohistochemical analysis showed P63 (−/+), CD68 (positive in multinucleated giant cells), SATB-2 (+/−), and Ki-67 positivity in approximately 10% of tumor cells (Figure 3).

**Figure 4:**
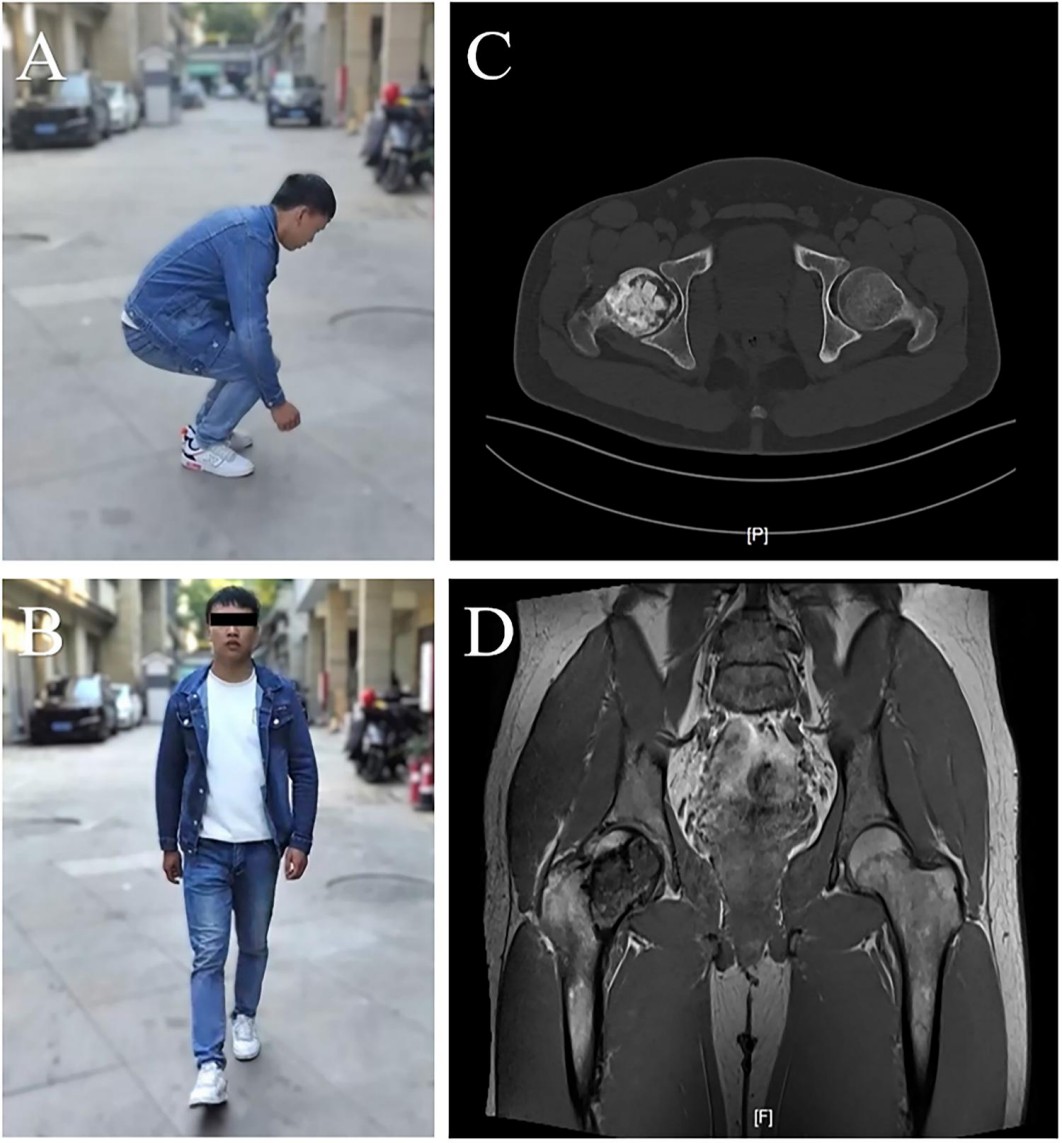
Functional recovery and radiographic follow-up at 9 months postoperatively. **(A)** Squatting activity without obvious limitation. **(B)** Walking activity without apparent restriction. **(C,D)** Follow-up imaging demonstrating no obvious resorption in the bone grafting area.

In the early postoperative period, the patient reported mild right hip pain accompanied by restricted motion. Given the extensive size of the lesion, strict limitation of weight bearing on the affected limb was maintained for 6 weeks postoperatively. Four weeks after surgery, the patient began a standardized hip rehabilitation program consisting of passive and active hip range-of-motion exercises, supplemented with physical therapy, including neuromuscular electrical stimulation, to prevent periarticular muscle atrophy. By 8 weeks postoperatively, the hip range of motion had returned to normal, with significant alleviation of pain. At the 9-month follow-up, the patient had resumed normal daily activities, with no apparent limitation in walking or squatting. Follow-up imaging demonstrated no obvious resorption of the mixed bone graft, and no clinical or radiographic evidence of local recurrence was observed (Figure 4).

## 3 Discussion

GCT involving the proximal femur is relatively uncommon, and its locally aggressive biological behavior, together with the anatomical proximity to the hip joint, poses substantial challenges for both clinical diagnosis and treatment (5). A notable feature of this case was that the lesion was located in the proximal femur and was relatively large in size, while the preoperative imaging findings lacked typical characteristic features, thereby increasing the complexity of preoperative assessment and treatment decision-making.

From a diagnostic perspective, GCT involving the proximal femur often presents with hip pain as the initial symptom, which is frequently refractory to rest and may be misinterpreted as a sports-related injury, leading to delayed diagnosis. In addition, its imaging features, such as cystic low-density lesions and marginal sclerosis, may overlap with those of bone cysts or other benign and borderline bone tumors (6, 7). Previous studies have also indicated that proximal femoral GCT is prone to misdiagnosis because of the lack of specific imaging characteristics (8). Therefore, in similar cases, increased awareness of potential borderline lesions is warranted, and a comprehensive evaluation integrating clinical presentation, imaging findings, and intraoperative frozen-section pathology is essential to reduce the risk of misdiagnosis and optimize surgical planning.

With regard to treatment strategies, extended intralesional curettage and marginal resection remain commonly adopted interventions for GCT without evident soft tissue involvement (9, 10). However, for lesions located in the proximal femur, there is still no consensus on optimal methods for postoperative bone defect reconstruction or functional preservation. Previous studies have suggested that combined grafting using autologous bone and artificial bone can be applied to the repair of complex bone defects and may offer advantages in terms of structural support and biocompatibility (11). In the present case, considering the deep location of the lesion, its proximity to the hip joint, and the patient's high functional demands, an arthroscopy-assisted strategy combining lesion curettage with mixed bone grafting was selected. Most reported treatments for proximal femoral GCT have relied on open surgical approaches, whereas reports on the application of arthroscopy-assisted techniques at this site remain relatively limited. This case demonstrates that arthroscopy assistance allows lesion curettage and bone grafting to be performed under favorable visualization, which may help minimize disruption to surrounding joint structures and soft tissues. In this patient, satisfactory short-term clinical and functional recovery was achieved, providing a technical approach that may serve as a reference for minimally invasive management of proximal femoral GCT.

Mechanical complications after extended intralesional curettage for GCT are not uncommon. A retrospective study reported that, at 5 years postoperatively, mechanical complications were the second most common cause of surgical revision after local recurrence (12). Nevertheless, compared with segmental resection, extended curettage has been associated with a lower rate of long-term mechanical complications and offers a more distinct advantage in preserving joint function (13). The femoral head and neck are critical load-bearing structures for force transmission, and GCTs arising in this region are associated with a substantially higher risk of mechanical complications than lesions located in other long bones. Previous studies have suggested that when the maximum diameter of a femoral neck lesion exceeds 50% of the femoral neck width, its mechanical strength may be compromised (14). In the present case, the femoral calcar remained intact without obvious invasion, and no pathological fracture was identified preoperatively. In addition, satisfactory defect reconstruction was achieved intraoperatively using mixed bone grafting, and protected weight bearing was planned after surgery. Considering these factors, prophylactic internal fixation was not performed. Another consideration was that internal fixation might increase the difficulty of future revision procedures in the event of recurrence or mechanical failure. Nevertheless, for extensive proximal femoral GCTs involving critical load-bearing structures such as the femoral neck, the greater or lesser trochanter, or the femoral calcar, prophylactic internal fixation should be strongly considered.

Given that recurrence rates of GCT vary according to anatomical location, with local recurrence rates of long bone lesions reported to be as high as 16.2% (15), regular postoperative imaging follow-up remains essential even after favorable initial outcomes. Surveillance incorporating local imaging and chest CT is recommended to facilitate early detection of local recurrence or distant metastasis, thereby improving long-term prognosis.

## 4 Conclusion

In summary, this case highlights the clinical challenges associated with the diagnosis and management of giant cell tumor of the proximal femur, and suggests that arthroscopy-assisted lesion curettage combined with mixed bone grafting achieved satisfactory short-term clinical and functional outcomes in this patient, which may provide a reference for the management of similar cases.

## Data Availability

The original contributions presented in the study are included in the article/Supplementary Material, further inquiries can be directed to the corresponding author.
